# *Mycobacterium avium* ssp. *paratuberculosis* and Crohn’s Disease—Diagnostic Microbiological Investigations Can Inform New Therapeutic Approaches

**DOI:** 10.3390/antibiotics13020158

**Published:** 2024-02-05

**Authors:** John M. Aitken, Jack E. Aitken, Gaurav Agrawal

**Affiliations:** 1 Otakaro Pathways Ltd., Innovation Park, Christchurch 7675, New Zealand; 2 Division of Diabetes & Nutritional Sciences, Franklin-Wilkins Building, King’s College London, London SE1 9NH, UK; gaurav.agrawal@kcl.ac.uk

**Keywords:** Crohn’s disease, Crohn’s dietary treatment, *Mycobacterium paratuberculosis*, microbiome, cell-wall-deficient mycobacteria, IBD treatment

## Abstract

*Mycobacterium avium* ssp. *paratuberculosis* (MAP) is the cause of Johne’s disease (JD), which is a chronic infectious gastrointestinal disease of ruminants and is often fatal. In humans, MAP has been associated with Crohn’s disease (CD) for over a century, without conclusive evidence of pathogenicity. Numerous researchers have contributed to the subject, but there is still a need for evidence of the causation of CD by MAP. An infectious aetiology in CD that is attributable to MAP can only be proven by bacteriological investigations. There is an urgency in resolving this question due to the rising global incidence rates of CD. Recent papers have indicated the “therapeutic ceiling” may be close in the development of new biologics. Clinical trial outcomes have demonstrated mild or inconsistent improvements in therapeutic interventions over the last decades when compared with placebo. The necessity to revisit therapeutic options for CD is becoming more urgent and a renewed focus on causation is essential for progress in identifying new treatment options. This manuscript discusses newer interventions, such as vaccination, FMT, dietary remediation and gut microbiome regulation, that will become more relevant as existing therapeutic options expire. Revisiting the MAP theory as a potential infectious cause of CD, rather than the prevailing concept of an “aberrant immune response” will require expanding the current therapeutic programme to include potential new alternatives, and combinations of existing treatments. To advance research on MAP in humans, it is essential for microbiologists and medical scientists to microscopically detect CWDM and to biologically amplify the growth by directed culture.

## 1. Introduction

Johne’s disease (JD) was first described by Johne and Frothingham in 1895 and was thought to be a form of bovine tuberculosis. The association of JD with *Mycobacterium avium* ssp. *paratuberculosis* (MAP) was made by Bang in 1906, and MAP was eventually successfully cultured by Fredrick Twort in 1912 on solid media supplemented with an extract of dried *Mycobacterium phlei*, glycerine–saline and egg [1]. Following quickly after the discovery that MAP was able to be grown, Twort began research on vaccine trials for JD. Reliable new methods for the bacteriological confirmation of MAP in JD-infected animals became available and accelerated veterinary and microbiological research into JD. By 1920 the widespread distribution of MAP globally in ruminants was able to be confirmed.

Ruminant animals with JD are cachectic and malnourished, with MAP infection detrimentally affecting body mass, milk production and reproduction. Diagnostic tests for the laboratory diagnosis of MAP infection in animals include mycobacterial culture, polymerase chain reaction (PCR), and enzyme-linked immunosorbent assay (ELISA). In animals, JD is often fatal if undiagnosed. The time taken for the detection of an infected animal in a herd is between 2–4 years, depending on the first appearance of clinical symptoms and the time taken for the detection of MAP in cultured samples. In ruminant animals, a diagnosis of JD is followed by culling the infected animal to prevent transmission of MAP to other members of the herd.

In humans, the association of MAP with Crohn’s disease has long been conjectured, but methods for the detection, culture and diagnosis of MAP infection have not been available. The primary reason for this absence has been the inability to reliably detect cell-wall-deficient mycobacterial (CWDM) forms of MAP in human blood and tissue samples from patients with CD. The detection of MAP as a CWDM form in patients with CD has been rare and has required extended incubation [2,3].

CWDM, sometimes referred to as “L forms” or spheroplasts, are bacteria devoid of a cell wall. The difficulties with the isolation of MAP in the CWDM form in humans are sometimes cited as reasons for the infrequency of MAP being found in samples from patients with CD. The ability to culture MAP from humans in the CWDM form enables animal studies and whole genome sequencing (WGS). For this to happen, reliable culture media for CWDM and easily performed stains will be required in order to confirm the presence of MAP in the laboratory, as happened with JD infections over 100 years ago.

## 2. The Pathogenic Potential of MAP

MAP is a member of the *Mycobacterium avium* complex (MAC), a group of mycobacteria formed around taxonomic and phenotypic traits, with some common features and clear differences. The taxonomy of members of MAC is based on various factors, including opportunistic pathogenicity of the organisms in specific animal hosts, dependence on mycobactin for growth, replication times, temperature ranges for growth in culture, and genome analyses. The inclusion of MAP within MAC has been debated and there are arguments for revising the classification of MAP as a subspecies of *Mycobacterium avium*. Some authors argue that MAP should be more accurately identified as *Mycobacterium paratuberculosis*. Factors influencing the taxonomy of MAP include the virulence potential of MAP in ruminant animals, and the increasing occurrence of acid-fast bacillary MAP infections in humans, subsequent to interaction with MAP-infected bison in India [4].

Partly because of the classification of MAP as a member of MAC, it has been proposed that the pathogenic potential of MAP in humans is in line with other members of MAC—that of an environmentally acquired, opportunistic pathogen. Opportunistic mycobacterial pathogens are seldom able to cause significant disease in humans with immunocompetent immune systems. In animals, particularly ruminants, MAP is pathogenic but in humans, the pathogenic range and infectious potential of MAP are largely unknown. However, there is growing global research interest in the role of MAP in association with human and animal diseases [5]. The decision to assign MAP to MAC should be reconsidered given the zoonotic potential of the organism and the dwindling availability of diagnostic tests and therapeutics for CD [6,7,8,9].

## 3. Isolation of MAP in Humans

MAP has the longest replication time of any opportunistically pathogenic Mycobacterium species (>24 h) resulting in extended incubation times for detection of growth.

Diagnostic medical laboratories using automated mycobacterial detection systems to culture opportunistic mycobacteria from human samples will only rarely isolate MAP from human samples. Such systems are optimised for the detection of MTBC but the short incubation times, from 6 to 8 weeks, that are suitable for isolation of MTBC, are unsuitable for some members of MAC—including MAP. These require extended incubation at varied temperatures and specific media supplementation.

Reports of the successful isolation of MAP in the bacillary form from human samples indicate that 12 to 18 months incubation is needed for detection.

In lieu of the culture detection of MAP in human samples, researchers have opted for polymerase chain reaction (PCR) to detect unique MAP sequences. The most common DNA sequences used for detection of MAP in samples are the insertion sequence IS900 and the F57 sequence element. Using PCR for IS900, carriage of MAP has been detected in both healthy controls and patients diagnosed with CD. PCR detection of MAP (IS900) in healthy patients has been widely reported, but only occasionally explained. One theory holds that viable MAP are widely distributed in the environment and various foodstuffs, (milk, water, and meat) and therefore transient intestinal colonisation is possible. Another possible explanation is the phenomenon of “environmental vaccination”.

This term was first proposed by Abrahams in 1970, as the theory of “original mycobacterial sin” in relation to primary mycobacterial contact and carriage [10].

This theory explores how initial contact by the host with a *Mycobacterium* species influences subsequent antigenic responses elicited by secondary exposure of the same host to other Mycobacterium species later in life. The theory suggests that viable mycobacteria may be retained in the bloodstream to augment “immune memory”.

Studies of healthy individuals who had been vaccinated with Bacillus Calmette-Guerin vaccine (BCG) at birth have demonstrated that BCG was retained in a cell-wall-deficient state by the vaccinated subjects, and could be reisolated decades later [11,12]. The long-term carriage of viable BCG suggests the possibility that primary exposure to MAP in early childhood, through environmental sources, may similarly explain the high levels of MAP carriage found in healthy adults.

CWDM forms of MAP present additional technical problems for diagnostic medical laboratories using methods that are optimised for the detection of MTBC. To successfully detect MAP in clinical samples, modifications to existing methods, culture media, and stains are required.

Partly for the reasons outlined above, the cultivation of MAP in the bacillary form in diagnostic medical laboratories is uncommon, unless MAP is specifically cultured for. In 1984, Chiodini et al. reported the cultivation of uncharacterised cell-wall-deficient mycobacteria from patients presenting with Crohn’s disease. The isolates reverted to bacillary forms of MAP after 18 months incubation [13]. There have been reports of the spread of MAP in the bacillary form among animal attendants tending MAP-infected bison in India [8]. Sechi et al., have reported a high level of culture-positive MAP infections in Sardinian patients with CD and some controls [14].

In 2002 Richter et al. reported the isolation of a bacillary form of MAP from a patient with HIV infection [15]. Samples collected from the patient included intestinal biopsy tissues, bone marrow and liver punch biopsies; all showed the presence of numerous acid- fast bacilli. The authors described the difficulties in culturing the acid-fast bacilli in samples collected from the patient. Despite laboratory visualisation of the acid-fast bacilli, cultures using commercial mycobacterial culture media and in-house media were inconclusive. After two years, the authors considered the possibility of MAP infection. Mycobactin J was thereafter added to culture media, and MAP in the bacillary form was grown after four weeks. The identity of the isolates as *M. avium* ssp. *paratuberculosis* was confirmed by PCR. This case highlighted the difficulties faced by diagnostic microbiology laboratories in the culture of MAP, even when the identity of the acid-fast bacillary isolate is suspected.

In 2023, Estevinho et al., succeeded in isolating bacillary MAP after 18 months, using a commercial culture system [16].

## 4. The Importance of the Diagnostic Laboratory

Parts of the role of a diagnostic microbiology department include the detection and identification of potentially pathogenic organisms, carrying out isolation and susceptibility tests, and providing guidance to clinicians regarding therapies. In addition, the 21st century ‘One Health’ concept includes “front line” monitoring for emerging zoonotic health threats by diagnostic microbiology laboratories. These tasks are counterbalanced by constrained health resources that limit these surveillance activities [17].

There are few papers describing the isolation of MAP from human samples in commercial mycobacteria culture media [15]. Culture systems, such as the BD BACTEC™ MGIT™ automated mycobacterial detection system (MGIT), are optimised for the isolation of MTBC in the acid-fast bacillary (AFB) phase of the mycobacterial life cycle. However, MAP requires additional supplementation with mycobactin J, which is not readily available in many human diagnostic laboratories. In addition, the extended incubation times required for MAP growth are outside the recommended incubation times of most commercial automated systems [18]. The isolation of CWDM forms of MAP is extremely difficult and as well as mycobactin J supplementation, complex, high-nutrient media and specific conditions for growth are required [19]. Chiodini et al., Timms et al., and more recently Estevinho et al., took approximately 18 months to isolate MAP from human samples [2,3,16].

Using alternative staining techniques and culture media, we are able to detect the presence of CWDM in human blood and tissue samples within two months, if the subject is in an acute phase of the illness [20]. The variant of the Ziehl Neelsen (ZN) stain used by many laboratories is optimised for the detection of MTBC. We have found that different ZN methods are more reliable for NTM and CWDM. Decolourising solutions using ethanol should be avoided. Ehrich’s modification of the ZN decolouriser (1883) is suitable for the detection of CWDM in cultures [21]. The CWDM isolates found in association with CD are acid fast, but are not acid–alcohol fast, particularly if ethanol is used in the decolouriser.

MAP detection is an example of the difficulties faced by diagnostic medical laboratories presented with the reality of emerging bacterial pathogens. A similar circumstance applies to *Mycobacterium xenopi* which has a slow growth rate and an optimal temperature for growth at 42 °C, so cannot easily be detected in automated systems [22].

## 5. Cell-Wall-Deficient Mycobacteria in Crohn’s Disease

**Figure 1:**
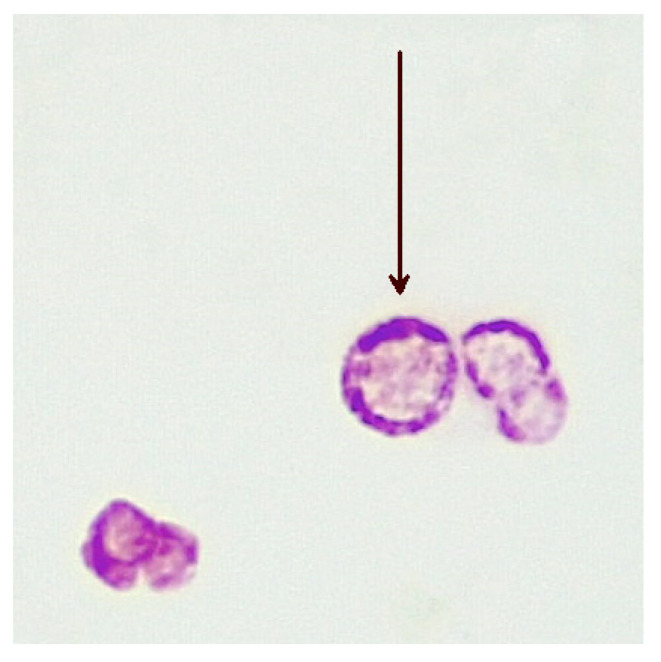
Deposition of mycolic lipids in the wall of the inner membranes of CWDMs (arrow) isolated from a patient with CD (×1000 magnification oil immersion ZN stain). Samples were positive for IS900.

In humans, the CWDM form of MAP remains elusive to bacteriologists. In vivo, CWDM are induced through enzymatic action which removes the outer membrane and the cell wall from the bacillary form. However, the cytoplasm remains intact and is encased by the remaining inner membrane. The inner membrane is semi-permeable and surrounds the cytoplasm, the region of the cell where mycolic acid is produced [23]. The presence of mycolic acid in proximity to the inner membrane explains why ZN staining of CWDM, with relevant changes in the decolouriser, shows acid-fastness in CWDM. Mycolic acids are highly antigenic and stimulate the production of tumour necrosis factor alpha (TNFα) in the host [24]. Mycolic lipid deposition in vitro is evident in the ‘donut’ morphology of the CWDM in cultures, where there is an accretion of mycolic lipids on the inner membrane. (Figure 1). This aggregation is a consequence of the inability of the CWDM to transport mycolic lipids from the inner membrane to the absent outer membrane. The precipitation of mycolic acid is a consequence of the elimination of the mmpL3 transporter [25,26] and the loss of the outer membrane.

**Figure 2:**
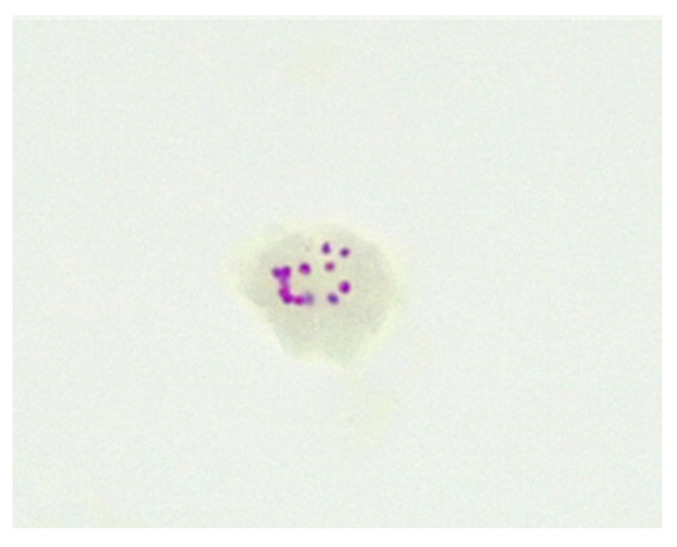
CWDM encased in biofilm from a patient with CD (×1000 magnification oil immersion ZN stain). Samples were positive for IS900.

Some antibiotics may penetrate the inner membrane of CWDM forms and enter the cytoplasm. However, the inner membranes of naturally occurring CWDM isolates are often enveloped in extracellular biofilm (Figure 2). In combination with biofilm, the deposition of mycolic lipids on the inner membrane wall presents challenges for the uptake of antimicrobial compounds. Despite these obstructions, antibiotics may infuse into the biofilm and thus have (limited) access to the inner membrane. Biofilm may also act as a ‘surrogate cell wall’ and retain the organism in the non-replicating persister (NRP) state. This biofilm matrix is also known to contain enzymes, such as nucleases [27]. The permeability of the inner membrane is bidirectional, so mycolic acid may also be present in the extracellular biofilm. The presence of NRP mycobacteria in the host subsequent to antimicrobial therapy, adverse environmental conditions or lack of sufficient nutrients and oxygen, may result in reduced effectiveness of antimicrobials [28] and require an extended period of effective therapy, because of the slow growth and metabolic activity of the organism.

**Figure 3:**
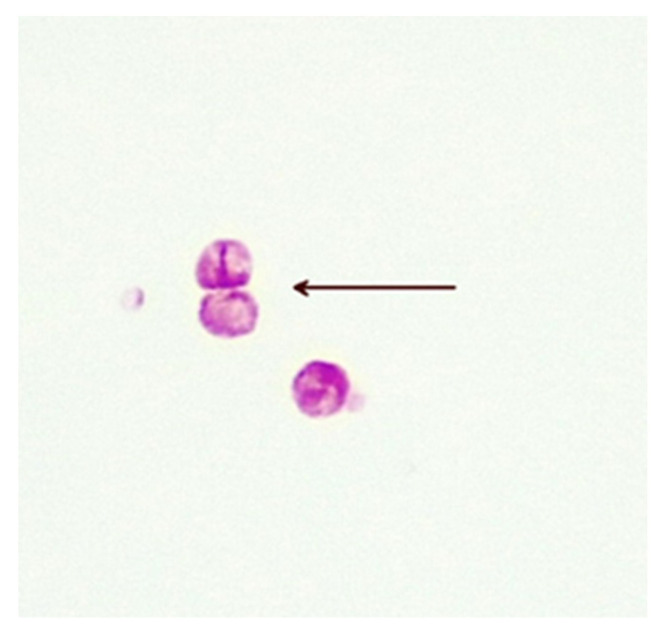
Bilateral division of CWDM forms (arrow) from a patient with CD (×1000 magnification oil immersion ZN stain). Samples were positive for IS900.

**Figure 4:**
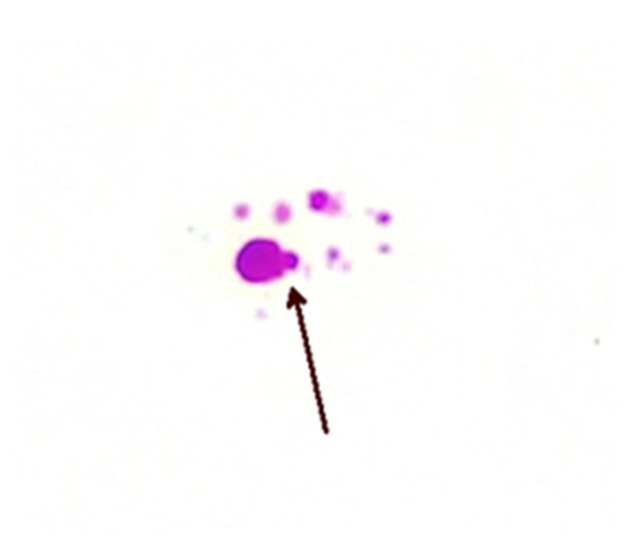
Bleb formation (arrow) on inner membrane from a patient with CD (×1000 magnification oil immersion ZN stain). Samples were positive for IS900.

The precise methods of replication of MAP CWDM are unclear, but there is evidence of a form of bilateral division (Figure 3). Microscopic examination of CWDM in cultures that suggest division are in contrast with the report that the *FtsZ* gene may be deficient in some intracellular MTBC [29]. There is also some evidence of blebs forming on the inner membranes [30], suggesting an alternative method of replication (Figure 4). Microscopy can demonstrate the presence of both bilateral division and “budding” in actively growing in cultures, though bilateral division is seen more commonly in culture.

## 6. Quantitation of Opportunistic Mycobacteria

An indication of the numbers of organisms present in a sample can be estimated microscopically, or by growth on solid media [31]. For opportunistic pathogens, such as MAP, that may be present in humans, quantitation is essential for the clinical assessment of the significance of isolates. Quantitation can also be a useful measure of therapeutic response. In the healthy subject, low numbers of MAC may be present without consequence. This phenomenon has been reported by researchers [32,33] and can confound diagnostic algorithms where mycobacterial infection is suspected. Use of PCR in those circumstances can be misleading, so multiple samples are required. Semi-quantitation of CWDM is a possibility when automated systems are used, provided new protocols and growth media for CWDM are developed. The automated detection of MTBC is time-dependent and that value relates to the numbers of organisms present in the original sample. The replication time for a strain of MTBC to be detected by the automated system may also be relevant. Prior to the use of PCR there was no requirement to quantify MTBC, except for study purposes and drug susceptibility research, as MTBC is an obligate pathogen. More recently, qPCR has been used to semi-quantitate numbers of MTBC organisms in samples [31].

## 7. Antimycobacterial Therapy for MAP Infection

### 7.1. Testing for Antimycobacterial Susceptibilities

Clinical trials assessing the effectiveness of antimycobacterial therapy in patients with CD, where MAP infection is suspected, cannot have MAP isolates prospectively tested for antibiotic resistance that may be present in the isolates prior to the commencement of the study. Given that some of these antimycobacterial therapies have been used for prophylaxis [34], the possibility exists that organisms may already be resistant to some of the antibiotics used in the combination therapies. Until reliable antimicrobial profiles are available, there will be some uncertainty about the performance of these combination therapies in individual patients.

### 7.2. Therapy for CD with Antimycobacterial Antibiotics

At present the following antibiotics are used for the treatment of infections of the gut and have shown short-term effectiveness: aminoglycosides, ciprofloxacin, metronidazole/tinidazole, Amoxicillin, cefuroxime, oral vancomycin, and fidaxomicin. In Crohn’s disease, the most commonly utilised antibiotics to specifically treat infection with MAP are Rifabutin, ciprofloxacin, clofazimine, clarithromycin, tinidazole and ethambutol. Treatment of MAP in humans has been built on an existing non-tuberculous mycobacterial antibiotic protocol. However, because of the limitations in culture methods, clinicians cannot know if the patient has a strain of MAP that is resistant to the treatment applied. One exception is the presence of the *rpoB* gene in mycobacteria, which confers resistance to rifamycins, including rifampicin [35]. Although some broad spectrum antibiotics are more specific for mycobacteria, they may still inhibit a broader range of intracellular bacteria in the human body [36]. The correct combination therapy is essential, along with knowledge of the clinical history of medications used on the patient. It is also important to consider inhibitory effects of other medications, and those that may induce resistance. Nonetheless, antimycobacterial therapy can be effective for suspected MAP-associated illnesses, such as CD, but rarely are they curative. Indeed, on removal of the therapy, patients often relapse—likely thought to be due to reactivation of CWDM.

## 8. Methods for Antimicrobial Susceptibility Testing

### 8.1. Molecular Detection of Resistance Genes

Next generation sequencing (NGS) has emerged as a technique for detecting the susceptibility of MTBC to a range of antimycobacterial drugs [37]. Its advantage is in its speed of detection, but limitations include the need for the isolate to be cultured in quantity. In the case of MAP, it may take weeks for the extraction of sufficient DNA for NGS.

### 8.2. Breakpoint Minimum Inhibitory Concentration (MIC)

This is an attractive option as the CWDM isolates can be introduced to a known concentration of antibiotics in a suitable growth medium, and the culture can then be monitored for growth/no growth. These techniques do not require NGS and are familiar to most microbiology medical scientists. The concentration of the antibiotic used in breakpoint testing is arrived at by establishing the concentration of the antibiotic that kills the target organism, and that is clinically compatible with the serum level achieved in the patient [38].

### 8.3. Alamar Blue

The Alamar Blue method monitors the metabolic activity of an isolate in a growth medium containing an antibiotic solution, and a control in the same growth medium without the antibiotic [39]. It has the advantage of being able to rapidly measure metabolism without having to monitor the replication rate of the test organism. Similar breakpoint concentrations are used in the Alamar Blue method as those used in the breakpoint method.

### 8.4. Pour Plate Counts

Our observations of CWDM in cultures have shown they are reliant on oxygen availability. The pour plate method will reduce available oxygen, favouring NRP.

### 8.5. Plate Counts Using Surface Inoculation

This is feasible using high nutrient agars but is yet to be trialled.

### 8.6. Animal Testing (Koch’s Postulates)

The development of an animal model has been explored on a number of occasions, using human isolates of MAP in the acid-fast bacillary (AFB) stage of the life cycle. These trials are more relevant if the CWDM strains of human MAP are used and the animal subjects are not transgenic. Feldman and Hinshaw have confirmed the in vivo effectiveness of streptomycin against MTBC in a guinea pig model, so a path exists to investigate prospective new therapies; however, again, this depends on the ability to isolate the CWDM forms of MAP from test subjects [40].

### 8.7. qPCR

The IS900 sequence has been demonstrated to be present in the MAP genome in 15 to 20 copies, when the genomes of animal isolates associated with JD are sequenced. The genome of the CWDM form found in Crohn’s disease has not yet been reliably sequenced, so numbers of copies of IS900 per cell are not known. A further problem with the semi-quantitative application of PCR for the monitoring of therapeutics [41,42] is the inability of the technique to readily differentiate between viable and dead organisms. Use of a control may provide a way of measuring comparative growth in cultures, with and without antimicrobial supplementation, as with breakpoint methods.

## 9. Potential Therapeutic Options for MAP Acquired CD

### 9.1. Dietary Regulation of the Gut Microbiome

Remediation of the gut microbiome is known to stabilise Crohn’s disease in some patients but therapeutic outcomes are inconsistent [43]. Dysbiosis precedes the acute phase of Crohn’s disease, presenting as a depletion of the gram-positive flora and an increase in gram-negative flora, particularly *Bacteroides fragilis* and *Escherichia coli*. Both organisms have the capability to produce toxins [44,45]. Remediation can be attempted through several interventions.

Induction of remission of Crohn’s disease through dietary intervention (EEN) is accepted as a valid therapy [46]. Regulation of diet to maintain remission and delay a flareup is more complex. Reintroduction of food after EEN may result in a rise in faecal calprotectin [47]. Maintenance diets suffer from the problem of low compliance, but inclusion of an anti-inflammatory programme can improve outcomes [48,49]. There are numerous dietary regimes which have been trialled and reported, but strong evidence for the efficacy of any regime is inconclusive. This is partly because of patient selection, concomitant medications, low compliance, and underuse of objective measures of inflammation [50]. Application of high-throughput sequencing will allow for greater understanding of the relationship between the host, the gut microbiome and diet in the patient with IBD. We have found that it is possible to test the in vitro response of CWD MAP organisms to amino acids and products of digestion absorbed into the bloodstream. L-tryptophan is capable of growth promotion of some CWDM, and is also negatively associated with IBD [51].

### 9.2. Probiotic Supplements

The use of probiotics for gut-associated disease is common, and research is generally supportive of a role for probiotics in IBD. However, much of the evidence relies on animal studies. Specific probiotics, such as *Escherichia coli* Nissle [52], have strong evidence of efficacy in gut-related infections and, more recently and partly as a result of high throughput sequencing studies, *Akkermansia* species, *Bifidobacterium* species and *Dietzia* species [53] have attracted research interest.

Probiotics are strain-specific, and commercial products vary considerably in composition and viability. Adding to uncertainty about the effectiveness of probiotics is the availability of many probiotics over the counter which may interfere with treatment response. Patients often self-medicate and may identify probiotic regimes that result in remission but are hesitant to report these to their healthcare providers. There is a need for better aligned definitive studies in this area to support the clinically directed use of probiotics [54,55].

### 9.3. Faecal Microbiome Transplant (FMT)

FMT may be effective in the therapy of Crohn’s disease, but the reasons for this are not well understood [56,57,58]. Several studies have reported that the use of sterile filtrates of FMT alone, may be effective in the control of C difficile infection [59,60]. Remediation of the gut microbiome by FMT can be short-lived in CD, and this response may mean that the primary cause is temporarily subdued by gut remediation, but not resolved. There is considerably more success with FMT when it is administered in combination with antimicrobial therapy [61].

### 9.4. Gut Sterilisation

Gut sterilisation was practiced in the 1970’s and 1980’s, when antibiotics were administered to reduce post-operative infections [62]. Gut sterilisation is now rarely performed, partly because of the selective effect on bacterial numbers [63], and partly because of concerns surrounding induction of bacterial resistance to antibiotics [64]. The use of antibiotics in preparation for FMT engraftment has been suggested, but the evidence for this is not supportive [65,66]. Remediation of the gut microbiome may be achievable if antibiotics are used in a targeted mode to reset the microbiome and increase gut bacterial diversity [67].

### 9.5. Bacteriophage Therapy

Bacteriophage therapy is a potential therapy in the post-antibiotic age that is a postulated consequence of bacterial evolution. Bacteriophages were first described by Twort in 1915 [68]. Phage therapy was first advanced as a potential antibacterial intervention a century ago for bubonic plague and intestinal infections. Despite the reports in the literature, bacteriophage therapy has only recently been seriously reconsidered as an emerging therapy for infectious diseases in western medicine. There are considerable barriers to acceptance, not least of which has been a rejection of the therapy by the infectious diseases community [68]. More recently, the emergence of the “superbugs” has caused concern because of their resistance to commonly used antimicrobials and this concern has focused attention on bacteriophage therapy.

Bacteriophages lyse bacteria through invasion of the cell wall or membrane. They infect the bacteria, and the bacteriophage is released during lysis to infect more bacteria until the infection is eradicated. Bacteriophages are highly specific, and only kill a specific organism. They are self-limiting; when all of the target bacteria are dead, the phage can no longer exist.

Bacteriophage therapy is a complex process, requiring libraries of bacteriophages which must be married to specific bacterial isolates. In brief, there are at least two approaches to the introduction of bacteriophages into the infected area. The first of these is the administration of a cocktail of different bacteriophages, and the second is the selection of a bacteriophage that is specifically matched to the target bacteria.

The regulatory framework around bacteriophage therapy requires formal approvals, and this may present challenges to regulators. Although bacteriophages are antibacterial, they do not fit within the current regulatory frame constructed for the use of antibiotics.

In an instance of CWDM, specifically in relation to MAP, there are serious barriers to progress, chiefly the structure of the target organism. MAP CWDM do not have cell walls, therefore the process of selection of a matching bacteriophage is complex. The CWDM must be isolated by culture before the selection process can be implemented. This is because there is no precedent for bacteriophage therapy, and therefore no predictable outcome. The inner membrane containing the cytoplasm of the CWDM is not identical to the mycobacterial outer membrane and cell wall of the parent mycobacterium. A starting point may be the D29 bacteriophage, which is able to lyse members of the *Mycobacterium genus* with intact cell walls. However, some researchers have hypothesised that the formation of CWDM may be a survival response to bacteriophage attack [69,70].

### 9.6. Infection Control

The control of the transmission of tuberculosis by infection control is an historical therapeutic intervention that prevents transmission of the infectious agent. Sanatoriums helped to control the transmission of tuberculosis in the community, as well as improving patient diets and general health. If MAP is an opportunistic pathogen infecting large numbers of hosts, then infection control will be important for eradication of the disease. As pointed out by Hermon-Taylor, the vector is most plausibly domestic livestock [71]. Control of livestock infection with MAP will eliminate the organism from the food chain.

### 9.7. Vaccination

Vaccination should be considered as part of a multi-modal approach to long-term therapy. The Bacillus Calmette–Guerin (BCG) vaccination for tuberculosis was first used in 1921 for protection against pulmonary tuberculosis and disseminated disease. Indirectly, BCG also provides some protection against *Mycobacterium leprae* and *Mycobacterium ulcerans* [72]. There have been studies indicating that the effect of BCG vaccination may extend to Crohn’s disease, but this effect has not been seen by researchers [73]. BCG is a live vaccine and that is factored in to any consideration of its use.

It is not known if certain genes are only expressed in certain stages of the mycobacterial growth cycle. CWDM may be capable of deleting or regulating gene expression to manage metabolism. This could affect vaccine targets. As an example, mycobacteria can metabolise both aerobically and anaerobically depending on environmental conditions [74]. Similar distinctions may impact on effective vaccination, particularly where living vaccines such as BCG are given. However, modern vaccines can now be used as a therapeutic strategy as well as a preventative one, with viral-vectored delivery systems able to stimulate T cells as well as B cells in the human [75,76]. It is unclear as to whether mRNA vaccines offer the same potential. Logically, they could be inferior, due to a lesser amount of the genetic component/antigens incorporated in the vaccine structure. It may also be that T cells are still not adequate to effectively eradicate the bacterium, as defective autophagy of macrophage activity and dysbiosis of the gut microbiome are also seen in Crohn’s Disease [76,77].

Vaccination provides the ability to enable long-term therapeutic immune responses to control the organism in the human body, but validation would require culture by semi-quantitative methods to optimise dosing and confirm the clinical efficacy of any vaccine. Assumptions could otherwise arise that may be inaccurate regarding the true mechanisms of action and effectiveness of the treatment. Mycobacteria can share genes and antigens and it is not known whether a general immune ‘boost’ would help inflammation and symptoms. All therapeutic trials have been hampered by the inability to culture and assess for MAP infection.

Once the whole genome sequences of human CWDM isolates are available for research purposes, it will be possible to allow precise engineering of human vaccines and personalised interventions that will be guided by genomic profiling of the CWDM. It could be that two differently constructed vaccines will be required for the different cell forms. Peptide and viral vectored vaccines are in production currently and have been trialled [78,79].

### 9.8. Immune Therapy

Gene therapy is the new frontier for immunologists. Interventions, such as the remediation of underlying aberrant immune responses, may be possible. Repurposing of orphan drugs, and drugs that have showed promise but have had development suspended, may be suitable for addressing immune deficiencies related to autophagy.

## 10. Discussion

Emerging stealth pathogens, particularly those of animal origin, are major challenges to medicine. The global presence of MAP in the environment, the food supply, in animals and in humans, presents challenges to the scientific research community, both in ascertaining the extent of the spread of MAP, and the impact (if any) of this on human and animal health. The proof of zoonotic transfer from ruminants to humans will be assisted by the laboratory detection and subsequent growth of the CWDM form of MAP. Meeting these challenges may require better diagnostic techniques, and new therapeutics.

In 2009, Hermon Taylor proposed a negative picture of the impact MAP would have, if it was to be a zoonotically transferred pathogen [71]. The predictions in his “Doomsday Scenario” paper could be occurring, and, despite ‘One Health’ concepts, there has been no definitive progress by the scientific community to acknowledge and act on this possible threat. In the instance of human-associated MAP infections, the task is made more difficult by the fact that MAP is a member of a complex (MAC) consisting of other opportunistic mycobacterial pathogens. Opportunistic infections with organisms such as MAP will require both laboratory and clinical awareness that MAP may be present in both CD, as well as in healthy subjects. MAP is not an obligate human pathogen like MTBC, so the laboratory approach to proof of infection is more nuanced. Proof of MAP infection does not lie solely in its presence but can only be confirmed with further evidence detailing the semi-quantitative detection of MAP when associated with the acute phase of the illness. The concept of an opportunistic pathogen versus an obligate pathogen means that the detection of MAP in culture is insufficient to be clinically definitive. One major difference between the transient carriage of an opportunistic mycobacterial pathogen, and the potential role of the same organism in an infectious process (commensal versus pathogenic), can be clarified through clinical presentation and the numbers of organisms present in cultures. If the numbers of organisms present in a patient sample are above those seen in samples from healthy individuals, then a pathogenic role for the organism should be factored into the clinical investigation [80].

CD is an inflammatory bowel disease. Considerable progress has been made over the last few decades in the suppression and control of inflammation, but the cause of the inflammatory reaction remains uncertain. Currently, there are concerning signals from the diagnostic and the therapeutic spheres with reference to the management of Crohn’s disease. The implementation of a precision medicine strategy, with clear pathways and algorithms, is necessary to achieve best outcomes for patients. This will require knowledge of individual patient histories, including predictors of response and disease characteristics [7,81]. Conversely, the recently published guidelines on the use of biomarkers in Crohn’s disease list three biomarkers for the routine monitoring of patients; calprotectin, erythrocyte sedimentation rate (ESR), and C-reactive protein (CRP) [9], with less reliance on colonoscopy. The treatment of the inflammatory response is complementary to the possibility of an infectious agent. Further understanding of this relationship could provide a new therapeutic approach.

The stealth lifestyle of MAP in the CWDM has been postulated and is now a strong consideration. Culture of the putative pathogen will answer the questions regarding infectivity, inflammation, and composition of MAP in the CWDM form. There are numerous potential reasons for the genesis of Crohn’s disease in the patient, but MAP will need to be included and investigated as a possible root cause. A pathogenic role for MAP in the patient diagnosed with CD can be neither confirmed nor denied without the demonstration of MAP presence in culture. If pathogenicity is proven, then therapeutic efficacy can be measured and tracked.

## References

[1] Twort F.W., Ingram G.L.Y., Hill L.E. (1997). A method for isolating and cultivating the mycobacterium enteritidis chronicæ pseudotuberculosæ bovis, Jöhne, and some experiments on the preparation of a diagnostic vaccine for pseudo-tuberculous enteritis of bovines. Proc. R. Soc. Lond. Ser. B Contain. Pap. A Biol. Character.

[2] Chiodini R.J., Van Kruiningen H.J., Thayer W.R., Coutu J.A. (1986). Spheroplastic phase of mycobacteria isolated from patients with Crohn’s disease. J. Clin. Microbiol..

[3] Timms V.J., Daskalopoulos G., Mitchell H.M., Neilan B.A. (2016). The Association of *Mycobacterium avium* subsp. *paratuberculosis* with Inflammatory Bowel Disease. PLoS ONE.

[4] Singh A.V., Chauhan D.S., Singh S.V., Kumar V., Singh A., Yadav A., Yadav V.S. (2016). Current status of *Mycobacterium avium* subspecies *paratuberculosis* infection in animals & humans in India: What needs to be done?. Indian J. Med. Res..

[5] Ekundayo T.C., Okoh A.I. (2020). Systematic Assessment of *Mycobacterium avium* Subspecies *paratuberculosis* Infections from 1911–2019: A Growth Analysis of Association with Human Autoimmune Diseases. Microorganisms.

[6] Mintz M.J., Lukin D.J. (2023). *Mycobacterium avium* subspecies *paratuberculosis* (MAP) and Crohn’s disease: The debate continues. Transl. Gastroenterol. Hepatol..

[7] Magro F., Moreira P.L., Catalano G., Alves C., Roseira J., Estevinho M.M., Silva I., Dignass A., Peyrin-Biroulet L., Danese S. (2023). Has the therapeutical ceiling been reached in Crohn’s disease randomized controlled trials? A systematic review and meta-analysis. United Eur. Gastroenterol. J..

[8] Singh A.V., Singh S.V., Singh P.K., Sohal J.S., Singh M.K. (2011). High prevalence of *Mycobacterium avium* subspecies *paratuberculosis* (‘Indian bison type’) in animal attendants suffering from gastrointestinal complaints who work with goat herds endemic for Johne’s disease in India. Int. J. Infect. Dis..

[9] Ananthakrishnan A.N., Adler J., Chachu K.A., Nguyen N.H., Siddique S.M., Weiss J.M., Sultan S., Velayos F.S., Cohen B.L., Singh S. (2023). AGA Clinical Practice Guideline on the Role of Biomarkers for the Management of Crohn’s Disease. Gastroenterology.

[10] Abrahams E.W. (1970). Original mycobacterial sin. Tubercle.

[11] Markova N., Slavchev G., Michailova L. (2015). Presence of mycobacterial L-forms in human blood: Challenge of BCG vaccination. Human. Vaccines Immunother..

[12] Dimova T., Terzieva A., Djerov L., Dimitrova V., Nikolov A., Grozdanov P., Markova N. (2017). Mother-to-newborn transmission of mycobacterial L-forms and Vδ2 T-cell response in placentobiome of BCG-vaccinated pregnant women. Sci. Rep..

[13] Chiodini R.J., Van Kruiningen H.J., Merkal R.S., Thayer W.R., Coutu J.A. (1984). Characteristics of an unclassified Mycobacterium species isolated from patients with Crohn’s disease. J. Clin. Microbiol..

[14] Sechi L.A., Scanu A.M., Molicotti P., Cannas S., Mura M., Dettori G., Fadda G., Zanetti S. (2005). Detection and Isolation of *Mycobacterium avium* subspecies *paratuberculosis* from intestinal mucosal biopsies of patients with and without Crohn’s disease in Sardinia. Am. J. Gastroenterol..

[15] Richter E., Wessling J., Lügering N., Domschke W., Rüsch-Gerdes S. (2002). *Mycobacterium avium* subsp. *paratuberculosis* Infection in a Patient with HIV, Germany. Emerg. Infect. Dis..

[16] Estevinho M.M., Cabeda J., Santiago M., Machado E., Silva R., Duro M., Pita I., Morais R., Macedo G., Bull T.J. (2023). Viable *Mycobacterium avium* subsp. *paratuberculosis* Colonizes Peripheral Blood of Inflammatory Bowel Disease Patients. Microorganisms.

[17] Reller L.B., Weinstein M.P., Peterson L.R., Hamilton J.D., Baron E.J., Tompkins L.S., Miller J.M., Wilfert C.M., Tenover F.C., Thomson R.B. (2001). Role of Clinical Microbiology Laboratories in the Management and Control of Infectious Diseases and the Delivery of Health Care. Clin. Infect. Dis..

[18] Mahomed S., Dlamini-Mvelase N.R., Dlamini M., Mlisana K. (2017). Failure of BACTEC^TM^ MGIT 960^TM^ to detect Mycobacterium tuberculosis complex within a 42-day incubation period. Afr. J. Lab. Med..

[19] Dane H., Stewart L.D., Grant I.R. (2023). Culture of *Mycobacterium avium* subsp. *paratuberculosis*: Challenges, limitations and future prospects. J. Appl. Microbiol..

[20] Aitken J.M., Phan K., Bodman S.E., Sharma S., Watt A., George P.M., Agrawal G., Tie A.B.M. (2021). A Mycobacterium species for Crohn’s disease?. Pathology.

[21] Bishop P.J., Neumann G. (1970). The history of the Ziehl-Neelsen stain. Tubercle.

[22] Piersimoni C., Nista D., Bornigia S., Gherardi G. (2009). Unreliable Detection of *Mycobacterium xenopi* by the Nonradiometric Bactec MGIT 960 Culture System. J. Clin. Microbiol..

[23] Bansal-Mutalik R., Nikaido H. (2014). Mycobacterial outer membrane is a lipid bilayer and the inner membrane is unusually rich in diacyl phosphatidylinositol dimannosides. Proc. Natl. Acad. Sci. USA.

[24] Sueoka E., Nishiwaki S., Okabe S., Iida N., Suganuma M., Yano I., Aoki K., Fujiki H. (1995). Activation of protein kinase C by mycobacterial cord factor, trehalose 6-monomycolate, resulting in tumor necrosis factor-alpha release in mouse lung tissues. Jpn. J. Cancer Res..

[25] Thouvenel L., Rech J., Guilhot C., Bouet J.-Y., Chalut C. (2023). In vivo imaging of MmpL transporters reveals distinct subcellular locations for export of mycolic acids and non-essential trehalose polyphleates in the mycobacterial outer membrane. Sci. Rep..

[26] Xu Z., Meshcheryakov V.A., Poce G., Chng S.-S. (2017). MmpL3 is the flippase for mycolic acids in mycobacteria. Proc. Natl. Acad. Sci. USA.

[27] Zang X., Dang G., Cai Z., Shao M., Tang Y., Cao J., Cui Z., Liu S. (2022). Extracellular DNase MAP3916c attacks the neutrophil extracellular traps and is needed for *Mycobacterium avium* subsp. *paratuberculosis* virulence. Vet. Microbiol..

[28] Quigley J., Lewis K. (2022). Noise in a Metabolic Pathway Leads to Persister Formation in *Mycobacterium tuberculosis*. Microbiol. Spectr..

[29] Chauhan A., Madiraju M.V.V.S., Fol M., Lofton H., Maloney E., Reynolds R., Rajagopalan M. (2006). *Mycobacterium tuberculosis* cells growing in macrophages are filamentous and deficient in FtsZ rings. J. Bacteriol..

[30] Lazenby J.J., Li E.S., Whitchurch C.B. (2022). Cell wall deficiency—An alternate bacterial lifestyle?. Microbiology.

[31] Marquetoux N., Ridler A., Heuer C., Wilson P. (2019). What counts? A review of in vitro methods for the enumeration of *Mycobacterium avium* subsp. *paratuberculosis*. Vet. Microbiol..

[32] Kuenstner J.T., Potula R., Bull T.J., Grant I.R., Foddai A., Naser S.A., Bach H., Zhang P., Yu D., Lu X. (2020). Presence of Infection by *Mycobacterium avium* subsp. *paratuberculosis* in the Blood of Patients with Crohn’s Disease and Control Subjects Shown by Multiple Laboratory Culture and Antibody Methods. Microorganisms.

[33] Juste R.A., Elguezabal N., Pavón A., Garrido J.M., Geijo M., Sevilla I., Cabriada J.L., Tejada A., García-Campos F., Casado R. (2009). Association between *Mycobacterium avium* subsp. *paratuberculosis* DNA in blood and cellular and humoral immune response in inflammatory bowel disease patients and controls. Int. J. Infect. Dis..

[34] Masur H. (1993). Recommendations on Prophylaxis and Therapy for Disseminated *Mycobacterium avium* Complex Disease in Patients Infected with the Human Immunodeficiency Virus. N. Engl. J. Med..

[35] Zaw M.T., Emran N.A., Lin Z. (2018). Mutations inside rifampicin-resistance determining region of *rpoB* gene associated with rifampicin-resistance in *Mycobacterium tuberculosis*. J. Infect. Public Health.

[36] Patangia D.V., Anthony Ryan C., Dempsey E., Paul Ross R., Stanton C. (2022). Impact of antibiotics on the human microbiome and consequences for host health. Microbiologyopen.

[37] Wu S.-H., Xiao Y.-X., Hsiao H.-C., Jou R. (2022). Development and Assessment of a Novel Whole-Gene-Based Targeted Next-Generation Sequencing Assay for Detecting the Susceptibility of *Mycobacterium tuberculosis* to 14 Drugs. Microbiol. Spectr..

[38] Ängeby K., Juréen P., Kahlmeter G., Hoffner S.E., Schön T. (2012). Challenging a dogma: Antimicrobial susceptibility testing breakpoints for *Mycobacterium tuberculosis*. Bull. World Health Organ..

[39] Carroll J., Douarre P., Coffey A., Buckley J., Cashman B., O’Farrell K., O’Mahony J. (2009). Optimization of a Rapid Viability Assay for *Mycobacterium avium* subsp. *paratuberculosis* by Using alamarBlue. Appl. Environ. Microbiol..

[40] Feldman W.H., Hinshaw H.C. (1948). Streptomycin: A Valuable Anti-tuberculosis Agent. Br. Med. J..

[41] Bard J.D., Lee F. (2018). Why Can’t We Just Use PCR? The Role of Genotypic versus Phenotypic Testing for Antimicrobial Resistance Testing. Clin. Microbiol. Newsl..

[42] Rolain J.M., Mallet M.N., Fournier P.E., Raoult D. (2004). Real-time PCR for universal antibiotic susceptibility testing. J. Antimicrob. Chemother..

[43] Rashed R., Valcheva R., Dieleman L.A. (2022). Manipulation of Gut Microbiota as a Key Target for Crohn’s Disease. Front. Med..

[44] Mirsepasi-Lauridsen H.C., Vallance B.A., Krogfelt K.A., Petersen A.M. (2019). *Escherichia coli* Pathobionts Associated with Inflammatory Bowel Disease. Clin. Microbiol. Rev..

[45] Sears C.L. (2001). The toxins of *Bacteroides fragilis*. Toxicon.

[46] Adamji M., Day A.S. (2019). An overview of the role of exclusive enteral nutrition for complicated Crohn’s disease. Intest. Res..

[47] Logan M., Clark C.M., Ijaz U.Z., Gervais L., Duncan H., Garrick V., Curtis L., Buchanan E., Cardigan T., Armstrong L. (2019). The reduction of faecal calprotectin during exclusive enteral nutrition is lost rapidly after food re-introduction. Aliment. Pharmacol. Ther..

[48] Heuschkel R. (2005). Synergy Between Immunosuppressive Therapy and Enteral Nutrition in the Management of Childhood Crohn’s Disease. J. Parenter. Enter. Nutr..

[49] Tokmak S. (2021). P012 Efficacy of Exclusive Enteral Nutrition and Weekly Adalimumab Combination in Crohn’s Patients with Fibroinflammatory Stenosis. Am. J. Gastroenterol..

[50] Mckirdy S., Russell R.K., Svolos V., Gkikas K., Logan M., Hansen R., Gerasimidis K. (2022). The Impact of Compliance during Exclusive Enteral Nutrition on Faecal Calprotectin in Children with Crohn Disease. J. Pediatr. Gastroenterol. Nutr..

[51] Nikolaus S., Schulte B., Al-Massad N., Thieme F., Schulte D.M., Bethge J., Rehman A., Tran F., Aden K., Häsler R. (2017). Increased Tryptophan Metabolism Is Associated with Activity of Inflammatory Bowel Diseases. Gastroenterology.

[52] Pradhan S., Weiss A.A. (2020). Probiotic Properties of *Escherichia coli* Nissle in Human Intestinal Organoids. mBio.

[53] Click R.E. (2011). Successful treatment of asymptomatic or clinically terminal bovine *Mycobacterium avium* subspecies *paratuberculosis* infection (Johne’s disease) with the bacterium Dietzia used as a probiotic alone or in combination with dexamethasone. Virulence.

[54] Prantera C. (2006). Probiotics for Crohn’s disease: What have we learned?. Gut.

[55] Vakadaris G., Stefanis C., Giorgi E., Brouvalis M., Voidarou C., Kourkoutas Y., Tsigalou C., Bezirtzoglou E. (2023). The Role of Probiotics in Inducing and Maintaining Remission in Crohn’s Disease and Ulcerative Colitis: A Systematic Review of the Literature. Biomedicines.

[56] Sokol H., Landman C., Seksik P., Berard L., Montil M., Nion-Larmurier I., Bourrier A., Le Gall G., Lalande V., De Rougemont A. (2020). Fecal microbiota transplantation to maintain remission in Crohn’s disease: A pilot randomized controlled study. Microbiome.

[57] Sarrabayrouse G., Landolfi S., Pozuelo M., Willamil J., Varela E., Clark A., Campos D., Herrera C., Santiago A., Machiels K. (2020). Mucosal microbial load in Crohn’s disease: A potential predictor of response to faecal microbiota transplantation. EBioMedicine.

[58] Boicean A., Birlutiu V., Ichim C., Anderco P., Birsan S. (2023). Fecal Microbiota Transplantation in Inflammatory Bowel Disease. Biomedicines.

[59] Kao D.H., Roach B., Walter J., Lobenberg R., Wong K. (2019). A51 effect of lyophilized sterile fecal filtrate vs lyophilized donor stool on recurrent clostridium difficile infection (rcdi): Preliminary results from a randomized, double-blind pilot study. J. Can. Assoc. Gastroenterol..

[60] Ott S.J., Waetzig G.H., Rehman A., Moltzau-Anderson J., Bharti R., Grasis J.A., Cassidy L., Tholey A., Fickenscher H., Seegert D. (2017). Efficacy of Sterile Fecal Filtrate Transfer for Treating Patients with Clostridium difficile Infection. Gastroenterology.

[61] Agrawal G., Clancy A., Huynh R., Borody T. (2020). Profound remission in Crohn’s disease requiring no further treatment for 3–23 years: A case series. Gut Pathog..

[62] Hodgson H.J.F. (1987). Review: Gut sterilization in inflammatory bowel disease. Aliment. Pharmacol. Ther..

[63] Croswell A., Amir E., Teggatz P., Barman M., Salzman N.H. (2009). Prolonged Impact of Antibiotics on Intestinal Microbial Ecology and Susceptibility to Enteric Salmonella Infection. Infect. Immun..

[64] Ramirez J., Guarner F., Bustos Fernandez L., Maruy A., Sdepanian V.L., Cohen H. (2020). Antibiotics as Major Disruptors of Gut Microbiota. Front. Cell. Infect. Microbiol..

[65] Singh P., Alm E.J., Kelley J.M., Cheng V., Smith M., Kassam Z., Nee J., Iturrino J., Lembo A. (2022). Effect of antibiotic pretreatment on bacterial engraftment after Fecal Microbiota Transplant (FMT) in IBS-D. Gut Microbes.

[66] Hassouneh R., Bajaj J.S. (2021). Gut Microbiota Modulation and Fecal Transplantation: An Overview on Innovative Strategies for Hepatic Encephalopathy Treatment. J. Clin. Med..

[67] Schwartz D.J., Langdon A., Sun X., Langendorf C., Berthé F., Grais R.F., Trehan I., Isanaka S., Dantas G. (2023). Effect of amoxicillin on the gut microbiome of children with severe acute malnutrition in Madarounfa, Niger: A retrospective metagenomic analysis of a placebo-controlled trial. Lancet Microbe.

[68] Brives C., Pourraz J. (2020). Phage therapy as a potential solution in the fight against AMR: Obstacles and possible futures. Palgrave Commun..

[69] Claessen D., Errington J. (2019). Cell Wall Deficiency as a Coping Strategy for Stress. Trends Microbiol..

[70] Ongenae V., Briegel A., Claessen D. (2021). Cell wall deficiency as an escape mechanism from phage infection. Open Biol..

[71] Hermon-Taylor J. (2009). *Mycobacterium avium* subspecies *paratuberculosis*, Crohn’s disease and the Doomsday scenario. Gut Pathog..

[72] Orujyan D., Narinyan W., Rangarajan S., Rangchaikul P., Prasad C., Saviola B., Venketaraman V. (2022). Protective Efficacy of BCG Vaccine against Mycobacterium leprae and Non-Tuberculous Mycobacterial Infections. Vaccines.

[73] El-Matary W., Yap J., Deora V., Singh H. (2018). Bacillus Calmette Guerin (BCG) Vaccine for Inducing and Maintaining Remission in Crohn’s Disease: A Systematic Review. J. Clin. Gastroenterol. Hepatol..

[74] Parrish N., Vadlamudi A., Goldberg N. (2017). Anaerobic adaptation of *Mycobacterium avium* subspecies *paratuberculosis* in vitro: Similarities to *M. tuberculosis* and differential susceptibility to antibiotics. Gut Pathog..

[75] Henderson P., Stevens C. (2012). The Role of Autophagy in Crohn’s Disease. Cells.

[76] Santana P.T., Rosas S.L.B., Ribeiro B.E., Marinho Y., de Souza H.S.P. (2022). Dysbiosis in Inflammatory Bowel Disease: Pathogenic Role and Potential Therapeutic Targets. Int. J. Mol. Sci..

[77] Singh V.P., Proctor S.D., Willing B.P. (2016). Koch’s postulates, microbial dysbiosis and inflammatory bowel disease. Clin. Microbiol. Infect..

[78] Abdellrazeq G.S., Elnaggar M.M., Bannantine J.P., Schneider D.A., Souza C.D., Hwang J., Mahmoud A.H.A., Hulubei V., Fry L.M., Park K.-T. (2019). A peptide-based vaccine for *Mycobacterium avium* subspecies *paratuberculosis*. Vaccine.

[79] Liu Y., Liao F. (2023). Vaccination therapy for inflammatory bowel disease. Hum. Vaccin. Immunother..

[80] Edwards B.D., Brode S.K., Mehrabi M., Marras T.K. (2022). Time to Positive Culture Detection Predicts *Mycobacterium avium* Pulmonary Disease Severity and Treatment Initiation. Ann. Am. Thorac. Soc..

[81] Zurba Y., Gros B., Shehab M. (2023). Exploring the Pipeline of Novel Therapies for Inflammatory Bowel Disease; State of the Art Review. Biomedicines.

